# Visualization and quantitation of epidermal growth factor receptor homodimerization and activation with a proximity ligation assay

**DOI:** 10.18632/oncotarget.19552

**Published:** 2017-07-25

**Authors:** Keiichi Ota, Taishi Harada, Kohei Otsubo, Akiko Fujii, Yuko Tsuchiya, Kentaro Tanaka, Isamu Okamoto, Yoichi Nakanishi

**Affiliations:** ^1^ Research Institute for Diseases of The Chest, Graduate School of Medical Sciences, Kyushu University, Higashi-ku, Fukuoka 812-8582, Japan; ^2^ Department of Respiratory Medicine, Japan Community Health Care Organization (JCHO) Kyushu Hospital, Kitakyushu City, Fukuoka, 806-8501, Japan; ^3^ Center for Clinical and Translational Research, Kyushu University Hospital, Higashi-ku, Fukuoka 812-8582, Japan

**Keywords:** epidermal growth factor receptor (EGFR), lung cancer, proximity ligation assay, receptor dimerization, tyrosine kinase inhibitor (TKI)

## Abstract

**Objectives:**

Activation of the epidermal growth factor receptor (EGFR) results from receptor homodimerization and autophosphorylation and confers sensitivity to tyrosine kinase inhibitors in some tumors. However, the visual detection and quantitation of activated EGFR in the clinical setting has not been established.

**Materials and Methods:**

A proximity ligation assay (PLA) was applied to detect EGFR homodimers in non–small cell lung cancer (NSCLC) cell lines and tissue specimens.

**Results:**

PLA signals corresponding to EGFR homodimers were higher in NSCLC cell lines and tissue specimens positive for activating *EGFR* mutations than in those wild type (WT) for *EGFR*. Stimulation with EGF in NSCLC cells WT for *EGFR* or forced overexpression of EGFR in Ba/F3 cells resulted in marked EGFR homodimerization. The extent of EGFR homodimerization appeared related to that of EGFR autophosphorylation in NSCLC cells WT for *EGFR*.

**Conclusion:**

PLA may provide a new tool for detection and quantitation of EGFR homodimers in NSCLC and other tumors.

## INTRODUCTION

The identification of activated forms of the epidermal growth factor receptor (EGFR) as oncogenic drivers in non–small cell lung cancer (NSCLC) has rendered this receptor a key target in precision medicine [1]. Activation of wild-type (WT) EGFR in response to ligand binding is mediated by receptor homodimerization and autophosphorylation. In contrast, activating mutations of EGFR present in some NSCLC tumors result in receptor dimerization in the absence of ligand and in constitutive activation of the receptor tyrosine kinase. Compared with standard platinum-based chemotherapy doublets, EGFR tyrosine kinase inhibitors (TKIs) such as gefitinib, erlotinib, and afatinib show more pronounced antitumor effects in NSCLC patients who harbor such activating EGFR mutations, which include in-frame deletions in exon 19 and an L858R point mutation in exon 21 [2–5]. Although the response rate of such patients to EGFR-TKIs is ∼80%, it follows that these drugs have no effect in ∼20% of mutation-positive patients. The response rate was only 8.9% in NSCLC patients WT for *EGFR* who received erlotinib after one or two prior chemotherapy regimens [6]. EGFR-TKIs also prolong survival in a subset of patients with colon, pancreatic, or head and neck cancer WT for *EGFR* [7–9]. Evidence thus suggests that the efficacy of such treatment varies among individuals regardless of *EGFR* mutation status and may also reflect EGFR activation not attributable to mutation.

Polymerase chain reaction (PCR)–based assays are usually adopted for detection of *EGFR* mutations [10]. However, a method for detection of EGFR activation that is not based on mutation identification has not been established in the clinical setting. We have now applied a proximity ligation assay (PLA) to visualize and quantitate EGFR homodimerization. We also examined the relation of EGFR dimerization determined by PLA analysis to EGFR autophosphorylation.

## RESULTS

### Detection of EGFR homodimers by PLA analysis

**Figure 1:**
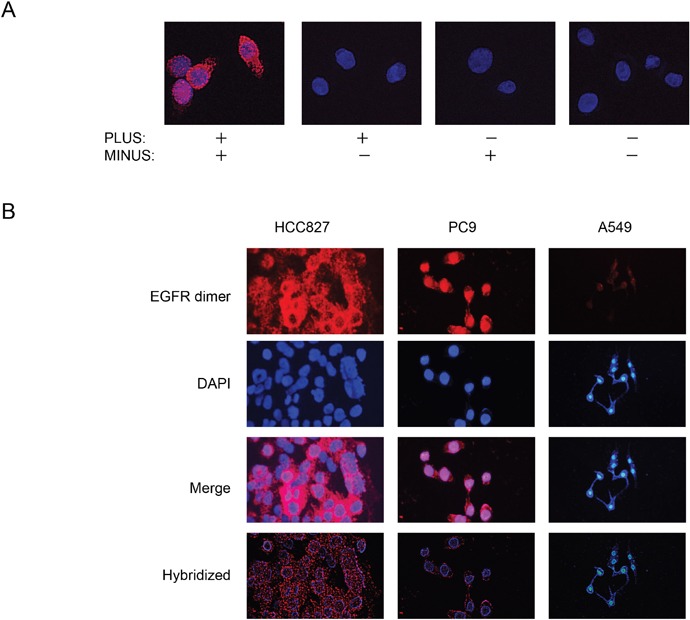
Detection of EGFR homodimers in NSCLC cell lines by PLA analysis **(A)** PLA analysis of PC9 cells performed in the absence or presence of PLUS and MINUS probes as indicated. Red signals corresponding to EGFR homodimers were detected only in the presence of both probes. Nuclei were stained blue with 4′, 6-diamidino-2-phenylindole (DAPI). **(B)** PLA analysis of *EGFR* mutation–positive (PC9, HCC827) or –negative (A549) NSCLC cell lines.

We first attempted to detect EGFR homodimers in seven NSCLC cell lines (Supplementary Table 1) positive or negative for activating *EGFR* mutations with the use of PLA probes derived from a monoclonal antibody to EGFR. PLA signals were detected in *EGFR* mutation–positive PC9 cells in a manner dependent on the addition of both PLUS and MINUS probes (Figure 1A), with annealing of the probes and consequent generation of the fluorescence signal indicating the presence of EGFR homodimers. PLA analysis also detected EGFR homodimers in *EGFR* mutation–positive HCC827 cells and to a much lesser extent in *EGFR*-WT A549 cells (Figure 1B), suggesting that the level of homodimerization is related to EGFR activation.

### Relation between EGFR homodimerization and autophosphorylation

**Figure 2:**
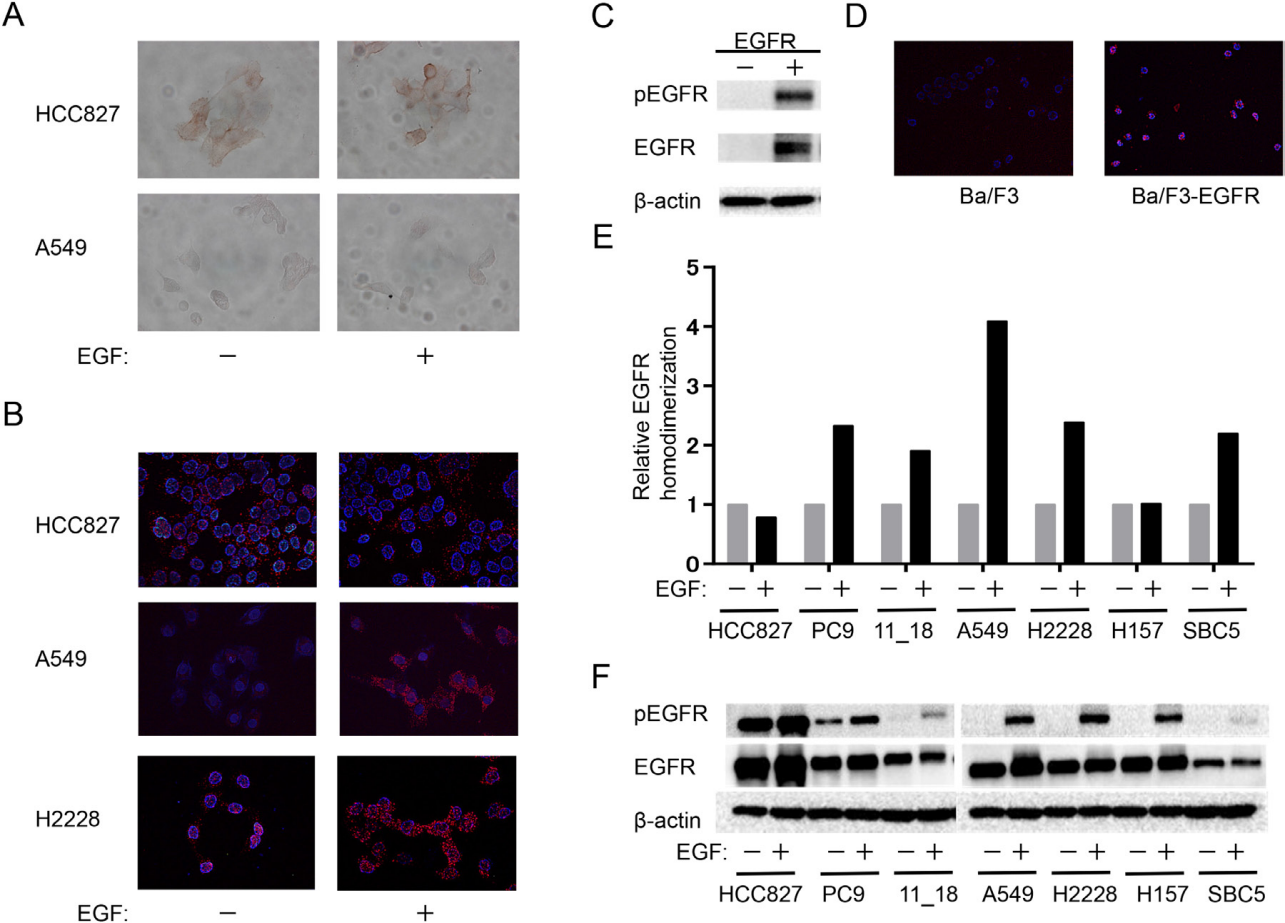
Relation between EGFR homodimerization and phosphorylation in NSCLC cell lines **(A, B)** The indicated cell lines were deprived of serum overnight, exposed (or not) to EGF (100 ng/ml) for 10 min, and then subjected either to immunohistochemistry with antibodies to EGFR (*A*) or to PLA analysis of EGFR homodimers (*B*). **(C, D)** Parental Ba/F3 cells or Ba/F3 cells transfected with an EGFR expression plasmid (Ba/F3-EGFR) and then cultured for 12 h were subjected either to immunoblot analysis of phosphorylated (p) or total forms of EGFR as well as of β-actin (loading control) (*C*) or to PLA analysis of EGFR homodimerization (*D*). **(E, F)** The indicated cell lines were deprived of serum and stimulated with EGF as in *A*. They were then subjected both to quantitative PLA analysis of EGFR homodimers (*E*) and to immunoblot analysis of EGFR phosphorylation (*F*). Data in *E* are expressed relative to the corresponding value for nonstimulated cells and are means from a representative experiment.

We next examined the effect of EGF on EGFR homodimerization in NSCLC cell lines. Whereas immunohistochemistry revealed no substantial effect of EGF on the pattern of EGFR expression in HCC827 or A549 cells (Figure 2A), PLA analysis showed that EGF induced EGFR homodimerization in A549 and H2228 cells (both of which are WT for *EGFR*) but not in *EGFR* mutation–positive HCC827 cells (Figure 2B). Furthermore, immunoblot and PLA analyses revealed phosphorylation (Figure 2C) and homodimerization (Figure 2D) of exogenous EGFR in transfected Ba/F3 cells. We then examined the relation between EGFR homodimerization (Figure 2E) and autophosphorylation (Figure 2F) in NSCLC cell lines positive (HCC827, PC9, 11_18) or negative (A549, H2228, H157, SBC5) for *EGFR* mutations. PLA analysis revealed that EGF induced a marked increase in the extent of EGFR homodimerization in all cell lines with the exception of HCC827 and H157. Similarly, immunoblot analysis showed that EGF markedly increased the extent of EGFR phosphorylation in most cell lines, although HCC827 showed a high basal level of such phosphorylation. These data thus suggested that the extent of EGFR homodimerization as determined by PLA analysis is related to the extent of EGFR phosphorylation in NSCLC cell lines.

### PLA analysis of NSCLC tissue

**Figure 3:**
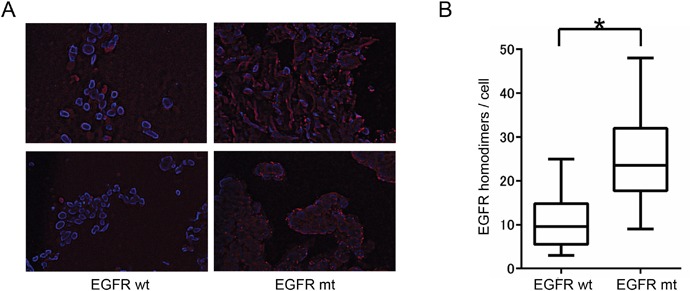
Relation between EGFR homodimerization and *EGFR* mutation in NSCLC tissue specimens **(A)** PLA analysis of EGFR homodimers in tumor tissue from two patients positive for *EGFR* mutations (EGFR mt) and two patients WT for *EGFR* (EGFR wt). **(B)** Box-and-whisker plots for the extent of EGFR homodimerization determined as in *A* for 15 patients with and 14 without *EGFR* mutations. *P < 0.05 (Mann-Whitney U test).

Finally, we applied the PLA method to NSCLC tissue obtained by transbronchial lung biopsy from 15 patients harboring *EGFR* mutations and 14 patients WT for *EGFR*. Consistent with the cell line data, the extent of EGFR homodimerization was significantly higher in tumors harboring *EGFR* mutations than in those WT for *EGFR* (Figure 3). These results thus indicated that the detection of EGFR homodimers by PLA analysis is also feasible for tissue samples from NSCLC patients.

## DISCUSSION

We have here demonstrated the detection of EGFR homodimers in NSCLC cells with a PLA.

PLA signals for EGFR homodimers tended to be higher in NSCLC cells harboring *EGFR* mutations than in those WT for *EGFR*. We further found that EGF increased EGFR homodimerization in association with induction of EGFR autophosphorylation in NSCLC cells WT for *EGFR*. Recent studies also found that PLA signals corresponding to EGFR-related interactions such as those between EGFR and GRB2 or between nonphosphorylated and phosphorylated forms of EGFR were higher in NSCLC cell lines positive for *EGFR* mutations than in those WT for *EGFR* [11–12]. EGFR belongs to the HER family, the members of which include EGFR (HER1), HER2, HER3, and HER4 and form heterodimers with each other as well as homodimers [13]. We also detected the formation of EGFR-HER2 heterodimers in PC9 cells by PLA analysis (Supplementary Figure 1A). Furthermore, we detected PLA signals corresponding to the EML4-ALK fusion protein in NSCLC cells harboring the *EML4-ALK* fusion oncogene (Supplementary Figure 1B), suggesting that this method will be applicable to the detection of other such fusion oncoproteins.

The PLA method also detected EGFR homodimerization in fixed tumor tissue from NSCLC patients. This analysis was performed with small NSCLC tissue specimens obtained by transbronchial lung biopsy, suggesting that the method should be applicable to the detection of EGFR homodimers in tissue microarrays. Given that EGFR-TKI therapy confers a survival benefit in some patients with colon, pancreatic, or head and neck tumors that are WT for *EGFR*, the amount of activated EGFR may be an important marker for the response to TKI therapy in such tumors. Whereas there is currently no commercially available antibody that is suitable for detection of EGFR activation by immunohistochemistry, our results now suggest that detection of EGFR homodimers in tissue samples by PLA offers an alternative approach to prediction of the response to EGFR-TKIs.

In conclusion, we have detected EGFR homodimers by PLA analysis in a quantitative manner in both NSCLC cell lines and tissue specimens obtained by transbronchial lung biopsy. The PLA signal for EGFR homodimerization appeared to be related to the extent of EGFR phosphorylation. PLA analysis may thus provide a new tool for visualization and quantitation of EGFR dimerization and activation.

## MATERIALS AND METHODS

### Cell culture and reagents

Cell lines were obtained and maintained as previously described [14], with the exception that SBC5 cells were provided by Japanese Collection of Research Bioresources. For PLA, cells were grown to 60% confluence on 12-mm-diameter cover glasses (Matsunami) placed in a 24-well plate (Greiner bio-one). EGF (Sigma-Aldrich) was dissolved in acetic acid (Wako) and stored at 4°C for up to 1 month.

### Patient specimens

Tumor specimens were obtained from 29 patients with lung adenocarcinoma (15 with an activating *EGFR* mutation, 14 WT for *EGFR*) who had undergone transbronchial lung biopsy between January 2007 and January 2016 at Kyushu University Hospital. *EGFR* mutations were identified by the peptide nucleic acid–locked nucleic acid PCR clamp method [10]. Specimens were fixed in 10% neutral buffered formalin, embedded in paraffin, sectioned, and treated as previously described [14]. The present study conforms to the tenets of the Declaration of Helsinki and was approved by the ethics review board of Kyushu University.

### *In situ* PLA and microscopy analysis

Cells were fixed for 20 min with 4% paraformaldehyde in phosphate-buffered saline (PBS) and permeabilized for 15 min with 0.2% Triton X-100 in PBS. An *in situ* PLA for detection of EGFR homodimers was performed with Duolink II PLA probes, Probemaker, and detection reagents (Olink Bioscience). Rabbit monoclonal antibodies to EGFR (Abcam) were conjugated to PLUS or MINUS PLA oligonucleotide arms with the use of Probemaker. Cells grown on cover glasses or tumor sections were incubated overnight at 4°C with the antibody-oligonucleotide complexes (PLA probes). Annealing of the PLUS and MINUS PLA probes occurs when two EGFR monomers are in close proximity, and repeat sequences in the annealed oligonucleotide complexes are amplified and then recognized by a fluorescently labeled oligonucleotide probe. PLA signals were detected with a Keyence BZ-8100 fluorescence microscope and were quantified with BZ Analyzer software (Keyence); 20 cells for each cell line and all cells in each image for tumor sections were examined for determination of the number of PLA signals per cell.

### Immunohistochemical staining

Cells were incubated overnight at 4°C with rabbit polyclonal antibodies to EGFR (Cell Signaling Technology), and immune complexes were detected with secondary antibodies, the streptavidin-biotin-peroxidase system, and 3, 3′-diaminobenzidine (Nichirei).

### Immunoblot analysis

Immunoblot analysis was performed as previously described [14]. Rabbit polyclonal antibodies to human Tyr^1068^-phosphorylated EGFR, to EGFR, and to β-actin were obtained from Cell Signaling Technology.

### Plasmid transfection

A Cell Line Nucleofector Kit V was obtained from Lonza. Ba/F3 cells (2 × 10^6^) were suspended in 100 μl of Nucleofector solution containing 2 μg of an expression vector for human EGFR [15] and were then subjected to electroporation according to program X-001 with a Nucleofector II Device (Amaxa Biosystems).

## SUPPLEMENTARY MATERIALS FIGURE AND TABLE


