# Supplementary material for: Visualization and quantitation of epidermal growth factor receptor homodimerization and activation with a proximity ligation assay
Source: Oncotarget. 2017 Jul 25;8(42):72127–32. doi: 10.18632/oncotarget.19552 (PMC5641116; doi:10.18632/oncotarget.19552)
Supplement: Supplementary file 1 [file oncotarget-08-72127-s001.pdf]

Visualization and quantitation of epidermal growth factor receptor homodimerization and activation with a proximity ligation assay

SUPPLEMENTARY MATERIALS

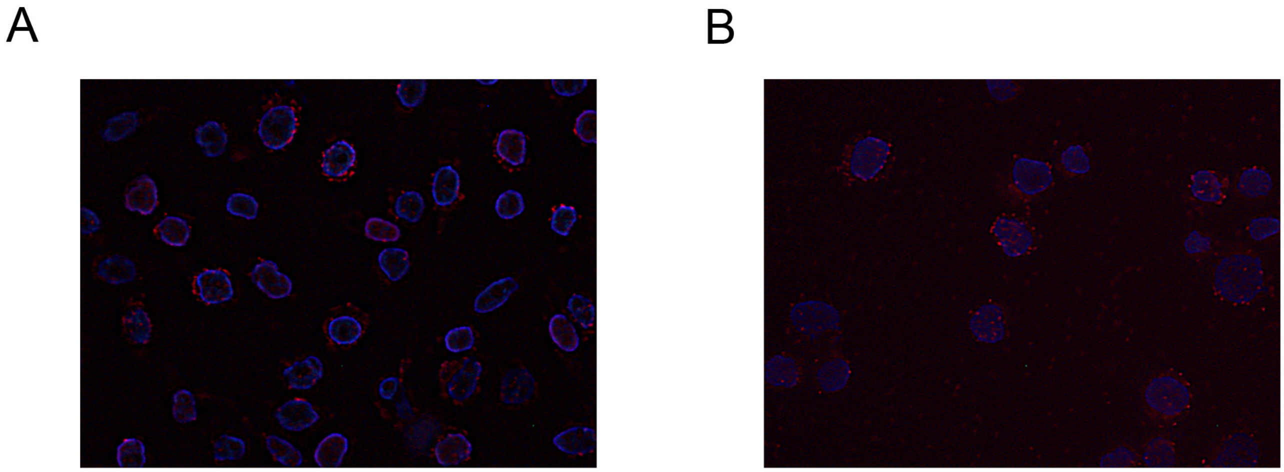

Supplementary Figure 1: (A) Detection of EGFR-HER2 heterodimers in PC9 cells by analysis with monoclonal antibodies to EGFR and to HER2 and a PLA kit (Olink Bioscience). **(B)** Detection of EML4-ALK in *EML4-ALK*–positive H3122 cells by analysis with monoclonal antibodies to EML4 and to ALK and a PLA kit (Olink Bioscience).

Supplementary Table 1: Lung cancer cell lines classified according to oncogene status and histology

Cell line	*EGFR* status	*ALK* status	Histology
HCC827	del(E746–A750)	Wild type	Adenocarcinoma
PC9	del(E746–A750)	Wild type	Adenocarcinoma
11_18	L858R	Wild type	Adenocarcinoma
H2228	Wild type	*EML4-ALK*	Adenocarcinoma
A549	Wild type	Wild type	Adenocarcinoma
H157	Wild type	Wild type	Adenosquamous cell carcinoma
SBC5	Wild type	Wild type	Small cell carcinoma
